# From Anxious to Reckless: A Control Systems Approach Unifies Prefrontal-Limbic Regulation Across the Spectrum of Threat Detection

**DOI:** 10.3389/fnsys.2017.00018

**Published:** 2017-04-07

**Authors:** Lilianne R. Mujica-Parodi, Jiook Cha, Jonathan Gao

**Affiliations:** ^1^Department of Biomedical Engineering, Stony Brook University School of MedicineStony Brook, NY, USA; ^2^Department of Psychiatry, Columbia University College of Physicians and SurgeonsNew York, NY, USA

**Keywords:** regulation, anxiety, sensation-seeking, addiction, limbic, fMRI, inferior frontal gyrus, Information Loop

## Abstract

Here we provide an integrative review of basic control circuits, and introduce techniques by which their regulation can be quantitatively measured using human neuroimaging. We illustrate the utility of the control systems approach using four human neuroimaging threat detection studies (*N* = 226), to which we applied circuit-wide analyses in order to identify the key mechanism underlying individual variation. In so doing, we build upon the canonical prefrontal-limbic control system to integrate circuit-wide influence from the *inferior frontal gyrus* (IFG). These were incorporated into a computational control systems model constrained by neuroanatomy and designed to replicate our experimental data. In this model, the IFG acts as an informational set point, gating signals between the primary prefrontal-limbic negative feedback loop and its cortical information-gathering loop. Along the cortical route, if the sensory cortex provides sufficient information to make a threat assessment, the signal passes to the ventromedial prefrontal cortex (vmPFC), whose threat-detection threshold subsequently modulates amygdala outputs. However, if signal outputs from the sensory cortex do not provide sufficient information during the first pass, the signal loops back to the sensory cortex, with each cycle providing increasingly fine-grained processing of sensory data. Simulations replicate IFG (chaotic) dynamics experimentally observed at both ends at the threat-detection spectrum. As such, they identify distinct types of IFG disconnection from the circuit, with associated clinical outcomes. If IFG thresholds are too high, the IFG and sensory cortex cycle for too long; in the meantime the coarse-grained (excitatory) pathway will dominate, biasing ambiguous stimuli as false positives. On the other hand, if cortical IFG thresholds are too low, the inhibitory pathway will suppress the amygdala without cycling back to the sensory cortex for much-needed fine-grained sensory cortical data, biasing ambiguous stimuli as false negatives. Thus, the control systems model provides a consistent mechanism for IFG regulation, capable of producing results consistent with our data for the full spectrum of threat-detection: from fearful to optimal to reckless. More generally, it illustrates how quantitative characterization of circuit dynamics can be used to unify a fundamental dimension across psychiatric affective symptoms, with implications for populations that range from anxiety disorders to addiction.

## Allostatic Regulation

### The Distinction between Injury and Disease

In the race to derive neuroimaging-based biomarkers for psychiatric disease, the principal challenge is not sensitivity but specificity. Most neuroimaging studies of psychiatric populations tend to implicate the same regions associated with emotion and affect (*amygdala, insula, prefrontal cortex, hippocampus, anterior cingulate*) described below as the *prefrontal-limbic control circuit*. Region-of-interest-based conceptualization of brain disorders has its historical origins in neurology, with its injury-based emphasis on lesions. Yet conceptually, the neuroimaging field might consider whether in fact psychiatric illnesses may share as much or more in common with non-brain-based diseases—such as diabetes, Cushing’s disease, heart disease and Grave’s disease—as with brain trauma.

**Figure 1:**
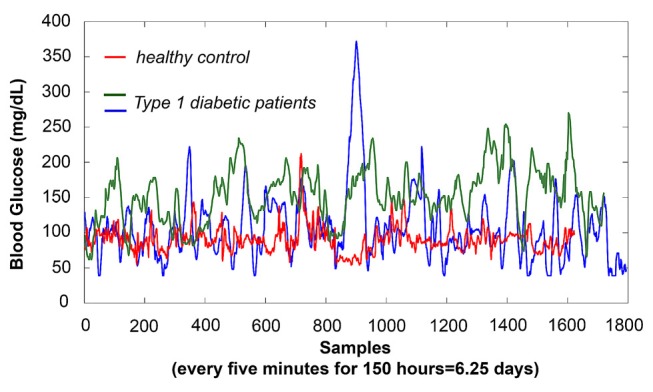
**Physiological negative feedback loops show outputs with characteristic dynamic signatures; dysregulation of the circuit causes a shift in dynamics that can be characterized by autocorrelation—either stronger or weaker, depending upon the type of dysregulation.** To illustrate a shift towards autocorrelation that is *stronger than optimal*, here we show three age and gender-matched subjects’ glucose time-series using an implantable MedTronic device, sampled every 5 min over 6.25 days. The glucose time-series produced by the Type 1 diabetic patients are more auto-correlated (self-similar, fractal) than those of the healthy control, in this case reflecting impaired negative feedback as glucose boluses trigger excitatory responses that are only weakly suppressed by insufficient insulin. As shown, detection sensitivity for differences in glucose amplitude varied dramatically during the day, as well as between days; thus, acquisition of random mean values over short periods of time (as typical for functional magnetic resonance imaging (fMRI) experiment, 10 min with TR = 2000 ms yields ~300 samples, which is roughly equivalent to 1 day of glucose measurements) would yield highly variable accuracy. However, even over this same period, patients showed markedly less complexity in their time-series than the healthy control. Using the Hurst exponent, in which maximum complexity is achieved at *H* = 0.5 with >*H* corresponding to stronger auto-correlation, our healthy control showed *H* = 0.68, with patients showing *H* = 0.82 and *H* = 0.83, respectively. A similar shift towards autocorrelation is seen in heart rate variability of heart disease patients, for whom the vagus nerve only weakly suppresses sympathetic excitatory responses. In contrast to the two above examples, in which circuit dysregulation is caused by changes in feedback strength, our neurobiological results suggest different, more complex, types of control circuit dysregulation caused by changes in gating and anatomical connectivity that affect feedback lag. These result in time-series with autocorrelations that are *weaker than optimal*, as shown in Figures 4, 6.

*Injuries* have singular onsets, with anatomically defined damaged loci. In contrast, *diseases* are inherently dynamic: resulting from dysregulation of the negative feedback loops that, in a healthy individual, function to maintain allostasis in the face of chaotic environmental inputs. These negative feedback loops are necessary because biological processes typically only are able to function within a narrow window of upper and lower limits for water, sodium, glucose, temperature, etc. Because the environment often includes perturbations that exceed those thresholds, the body maintains homeostasis by negative feedback loops that correct the system towards baseline. For example, an acute bolus of glucose, unopposed, would lead to a hyperglycemic coma. Therefore, the metabolic control circuit responds by secreting the hormone insulin, sending the system into postprandial reactive hypoglycemia. Because hypoglycemia is just as dangerous to the body as hyperglycemia, the metabolic control circuit then secretes a different hormone, glucagon, which releases glucose back into the bloodstream. In a healthy person, the negative feedback loop as whole functions as a damped oscillator, with multiple excitatory (e.g., glucose, glucagon, cortisol) and inhibitory (insulin) responses acting in series to maintain glucose within acceptable limits. In a person with diabetes, however, the same perturbation is inadequately controlled—leading to extreme oscillations between hyper and hypoglycemia (Figure 1).

The analogy to diabetes has several features with potential implications for psychiatry. First, *the same control circuit can be dysregulated in more than one way, with distinct etiologies, and resulting in divergent clinical features*. Type 1 diabetes is feed-forward problem: when glucose rises, insulin is not produced. Type 2 diabetes is a feedback problem: when insulin rises, glucose is not suppressed. Yet while the same components of the negative feedback loop that regulates blood sugar, glucose and insulin, are implicated in both, untreated Type 1 and Type 2 diabetics have distinct—and, in some cases, opposite—clinical features. The former are underweight, begin to show symptoms early in life, and have trouble regulating glucose because of an autoimmune disease that attacks the pancreas and therefore impairs insulin production. The latter are overweight, begin to show symptoms later in life, and have trouble regulating glucose because of a high-glycemic diet that chronically overtaxes, and over time desensitizes, insulin response. By extension, most psychiatric diseases may implicate the same prefrontal-limbic regions, yet differences in the type of dysregulation within the circuit may lead to markedly different clinical characteristics.

The second feature is that *dysregulation is not most sensitively characterized by amplitudes but by dynamics, and therefore is most clearly seen in response to perturbation*. A Type 1 diabetic patient, a Type 2 diabetic patient, an individual with pre-diabetes, and a healthy control can all show—under the right circumstances (for example, before or right after eating) completely indistinguishable glucose and insulin amplitudes. Instead, the dysregulation is most sensitively measured as the dynamic response of the system as a whole as it attempts to regulate to baseline in response to positive or negative perturbation^1^. This feature is relevant for psychiatry because nearly all neuroimaging studies focus upon either amplitude (contrast-based general linear model) or resting-state analyses. Neither is ideally suited for probing dysregulation, since the former is not optimized for characterization of dynamics, while the latter lacks perturbation. Thus, if psychiatric disease is—like other physiological diseases—dysregulatory, then standard neuroimaging approaches may be complemented by considering techniques (from engineering, physics and physiology) that were specifically designed to detect abnormalities in negative feedback loops. As a field, we may benefit from expanding our conceptualization of what it is that neuroimaging ought to be measuring.

The third feature is that *first onset of clinical symptoms typically do not mark the beginnings of dysregulation, but rather the end-stage of a chronic degenerative process*. Because dysregulation leads to larger excursions following perturbation, and those larger excursions in turn put greater stress on the control system to maintain allostasis, disease processes often trigger a vicious cycle that further degenerates over time. This feature implies that small degrees of dysregulation can be detected pre-symptomatically (as per the use of the glucose tolerance test to diagnose pre-diabetes), that they should be detected pre-symptomatically (to more easily correct a trajectory that is likely to become self-reinforcing), and that knowledge of dynamics at any given time can provide some degree of prediction with regard to future states. Schizophrenia (Lieberman et al., 1996; Marshall et al., 2005), major depressive disorder (Burcusa and Iacono, 2007), addiction (Dewit, 1996), and bipolar disorder (Joyce et al., 2016) are all psychiatric illnesses considered to show priming effects (each additional episode increases risk for future episodes); therefore, a better understanding of dysregulation during the prodrome may not only yield better treatment options, but may also provide needed insight into the timing and duration of periodic relapse.

### The Prefrontal-Limbic System as a Control Circuit

**Figure 2:**
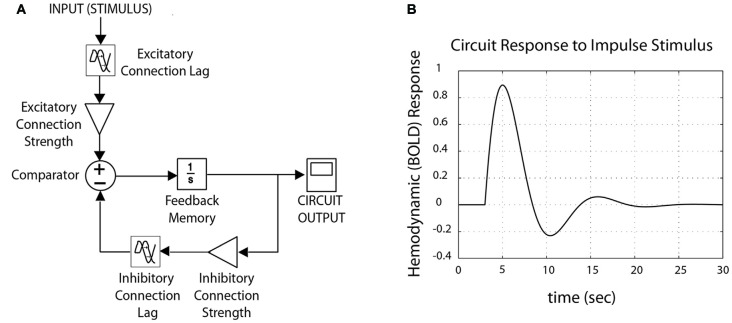
**Schematic control system, tuned for fMRI data. (A)** A schematic control system is structured as a negative feedback loop, with both excitatory and inhibitory components. Circuit-wide dynamics change as a function of lag and connection strength (“connectivity”) between nodes, resulting from variation in synaptic plasticity and/or neurotransmitter/receptor density. **(B)** Outputs from the model produce waveforms comparable to canonical hemodynamic response function typical for fMRI (here, shown for impulse stimulus).

**Figure 3:**
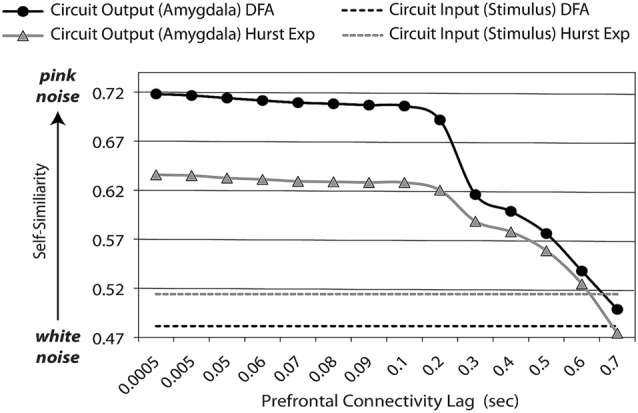
**Feedback lag affects dynamic (entropic) signatures of circuit outputs.** An adaptive prefrontal-limbic control circuit must be sufficiently supple to respond to the environment, yet sufficiently constrained to efficient return back to baseline. Optimization over these two properties gives rise to general dynamic signatures, which are evident not only with frequency-based methods (such as power spectrum scale invariance, PSSI), but those based upon autocorrelation: *Hurst exponents* (Hurst Exp) and *detrended fluctuation analysis* (DFA, which is used with non-stationary time-series). Assuming excitatory and inhibitory components are equal, with minimal lag in feedback, time-series dynamics are balanced (*pink noise*, *β* = 1, *Hurst Exp* = (1 + *β*)/2 = 1) between maximum complexity/chaos (*white noise*, *β* = 0, *Hurst Exp* = (1 + *β*)/2 = 0.5) and order. As the lag for feedback lengthens, it affects system output dynamics by reducing the amount of “memory” (auto-correlation) in the system; at very long lags, it is no longer operating as a closed circuit.

Physiological control circuits that maintain homeostasis do so by means of negative feedback loops (Figure 2). Negative feedback loops contain three basic conceptual elements: (1) *excitatory* components, which increase circuit outputs; (2)* inhibitory* components, which suppress circuit outputs; and (3) *feedback*, which allows circuit outputs to act as future circuit inputs. They may also include *gains* (which increase or decrease signal strength), *lags* (which affect signal propagation time), and *filters* (which let through some signal frequencies while blocking others). Formally, activation from excitatory and inhibitory components converge upon a *comparator*^2^, which compares one or more inputs, and applies a function to the result. Feedback provides “memory” within the circuit, which rather than resetting for every iteration of the cycle through the negative feedback loop, builds cumulatively upon previous values. Thus outputs of a feedback loop are not solely the product of circuit inputs, but also the product of previous circuit outputs; this feature introduces important nonlinearities that can have nontrivial effects over time (Figure 3).

The brain’s primary neural substrate for emotion regulation is the *prefrontal-limbic system* (Ledoux, 1996). In this model, derived primarily from rodent studies of Pavlovian threat conditioning, sensory stimuli are received as inputs and filtered for novelty via the *sensory thalamus*. Thalamic outputs are then routed along two pathways. The “low road” proceeds to the *amygdala*, which rapidly mounts an excitatory response. This is the most direct pathway, but in optimizing over speed, utilizes only low-resolution sensory data to make a preliminary determination of threat (Ohman, 2005; Ohman et al., 2007; Pessoa and Adolphs, 2010). In parallel, the “high road” proceeds to the *sensory cortex*, which acquires high-resolution sensory information. This sensory information is relayed to the prefrontal cortex and hippocampus, which together pattern-match (Leutgeb et al., 2007; Bakker et al., 2008; Nakashiba et al., 2012) sensory information to determine threat vs. safety. These (excitatory) cortical signals, in turn, relay to the *lateral* and *basolateral amygdala*. The boundary between these two amygdala sub-regions acts as a comparator with excitatory and inhibitory inputs, which together modulate amgydala outputs. These include outputs from the basolateral amygdala signal to the *nucleus accumbens*, and thus to the reward circuit, as well as outputs from the central medial amygdala signal to the *hypothalamus*, which then feeds into two other control circuits: the autonomic nervous system and the endocrine hypothalamic-pituitary-adrenal (HPA) axis. Both of these peripheral systems also feed back to the brain, creating nested control systems.

While there is general consensus that the prefrontal cortex contributes to arousal inhibition, different human studies have implicated different sub-regions, including the *dorsolateral prefrontal cortex* (DLPFC; Goldin et al., 2008), *ventromedial prefrontal cortex* (vmPFC; Roy et al., 2012), and inferior frontal gyrus (IFG; Aron et al., 2003; Hampshire et al., 2010). This disparity has several likely causes. First, rodent brains (upon which most of the basic neuroscience circuits were first defined) and human brains (upon which most of the psychiatric research has been conducted) are less clearly homologous in the prefrontal cortex than in other regions, which makes translation across species difficult. Second, even amongst humans, our methods for describing the same anatomical region across neuroimaging studies remain imperfect, given the computational challenges of accurately normalizing across brains. Finally, neuroimaging studies on emotion are heterogeneous, which might implicate different regions in the PFC depending upon the precise nature of the task and its demands.

### Beyond Human Brain Mapping: Quantifying “Regulation” Locally vs. Globally

To date, functional magnetic resonance imaging (fMRI) is generally used for human neuroimaging in essentially two ways: to infer brain activation maps (areas of differential hemodynamic response) and to infer brain connectivity between dyads (regions that are co-activated). Activation maps are inferred by statistical comparisons between experimental conditions or populations, revealing task-activated neuroanatomical areas (Poline and Brett, 2012). Newer connectivity-based techniques rely upon time-course cross-correlations between two voxels or anatomically-defined regions to infer connection strength (Stephan and Friston, 2010). This is true of resting-state study designs, which remove the subtraction element of fMRI analysis, but maintain dependence on identifying regions of interest (Greicius et al., 2003), as well as graph theoretic measures that quantify global connectivity features via correlation matrices (Bassett and Bullmore, 2006). What both activation and connectivity critically miss, however, is the dynamic concept of feedback—an essential feature of all control circuits, and therefore regulation.

If we can conceptualize healthy emotion regulation as a well-regulated control circuit, and pathological emotion regulation as a dysregulated control circuit, then we need to be able to define quantitatively what we mean by “well regulated” and “dysregulated”. Note again that all disorders of dysregulation, like diabetes, manifest clinical values that are neither necessarily higher nor lower value than healthy values at any given time. Rather, just as with hyper and hypoglycemia, they exhibit a dynamic of uncontrolled oscillations that lead to heightened and lowered values at different times^3^.

There are two fundamental approaches towards quantifying system-wide regulation dynamics: *local* and *global*. In the most simplistic local approach, we identify the dynamics of the system as a whole as a function of the dynamics of its individual parts. For example, in Figure 2, by identifying the strength (gain) of each wire connecting the nodes, we could thus weight the excitatory relative to inhibitory components, and express them as a ratio. This approach has been used in the power spectrum density and principal dynamic modes (Chon et al., 2006; Zhong et al., 2006, 2007) techniques in Heart Rate Variability (HRV) (1996), quantifying autonomic regulation as the ratio of excitatory (sympathetic) over inhibitory (parasympathetic) components. Analogously for neuroimaging, excitatory and inhibitory connection strength between nodes can be defined using connectivity techniques such as dynamic causal modeling (Stephan and Friston, 2010) and Granger causality (Roebroeck et al., 2005); in theory, ratios between them could be used to quantify regulation. However, excitatory/inhibitory ratios omit the self-referentiality and lag aspects of feedback, both critical components of control systems and their nonlinear dynamics.

In contrast, the global approach characterizes the dynamics of the system as whole rather than as a function of its individual parts. In the last few years, our group (Rădulescu and Mujica-Parodi, 2008, 2014; Tolkunov et al., 2010; Rubin et al., 2013; Cha et al., 2016) and others (He et al., 2010; Lai et al., 2010) have begun to use *nonlinear complexity* methods (such as power spectrum scale invariance (PSSI), detrended fluctuation analysis (DFA), Hurst exponents, Lyaponov exponents, and Shannon entropy), in conjunction with fMRI, to probe intact negative feedback loops in the brain. These methods, first applied to physiology in the context of the autonomic nervous system (Kurths et al., 1995; Ho et al., 1997; Mäkikallio et al., 1997; Mujica-Parodi et al., 2005; Hu et al., 2009), exploit the fact that negative feedback loops provide unique dynamic signatures, which are disrupted when the system deviates from efficient homeostatic regulation (Gisiger, 2001; Rădulescu and Mujica-Parodi, 2014).

Using modeling and simulations, we have previously shown (Rădulescu and Mujica-Parodi, 2014) that the outputs of brain-like negative feedback loops create a balance of frequencies that follow a power law; i.e., are *scale-invariant*, following *S(f) ∝ f^−β^*. As a control system increases feedback, the circuit’s output at any given time is increasingly influenced by the same circuit’s output at previous times—the timing of which is a function of feedback lag, as well as the number of previous cycles. This increased “memory” within the system increases autocorrelation within the time-series, and therefore reinforces the lower-end of the frequency spectrum. When excitatory and inhibitory components are perfectly balanced, with sufficient lag to permit a response but with feedback that triggers fast enough to suppress it, the power-law shows a distribution of frequencies known as *1/f^β^*, *β* = 1 or *pink noise*. Pink noise describes a frequency distribution poised at the midpoint between chaos (*β* = 0; equal power over all frequencies: *white noise*) and order (*β* > 2; zero power over all frequencies except for one: *black noise*). As time-series shift away from pink noise towards black noise, negative feedback loops become more than optimally constrained, and thus are unable to efficiently respond to their environments. As time-series shift away from pink noise towards white noise, negative feedback loops are less than optimally constrained, and thus are unable to efficiently recover from perturbation. Optimal negative feedback loops are balanced at what is sometimes described within physical systems (Bak et al., 1987) as a meta-stable “critical point.” Within biological systems this meta-stable state is metabolically efficient, requiring the least amount of energy both to respond to perturbation as well as to return to baseline. This metabolic advantage suggests that evolution may underlie pink noise dynamics ubiquitous in biological and ecological systems (for review, see Gisiger, 2001).

Complexity methods are unique in that they are capable of describing system-wide behavior of (nonlinear) feedback loops. As such, they excel as diagnostic tools because results provide information about the locus and type of dysregulation (Rădulescu and Mujica-Parodi, 2014). Their primary disadvantage is that they yield minimal information about a system’s structure, and cannot provide simulations that predict future trajectories. One might think that the local approach is preferred, simply because—at first glance—it seems to provide more information about the system. And—at first glance—this is true, since the global approach is agnostic towards the individual components of the system, which exist essentially as a black box. However, in any complex system in which all of the individual components and their dynamics are not fully known, local approaches generally fail by virtue of incomplete or incorrect specification of the underlying neural systems. Because they rely upon the system’s architecture, they are vulnerable to underestimating that structure, or to getting it wrong. Primarily for this reason, global approaches—which assume less—have been found to provide a more accurate diagnostic for autonomic dysregulation (Kurths et al., 1995; Voss et al., 1995; Ho et al., 1997; Mäkikallio et al., 1997, 1998, 2002; Hu et al., 2009). Therefore, our strategy will be to start with a global approach to identify, in an agnostic manner unbiased by *a priori* knowledge of the brain, key nodes of the prefrontal-limbic system and its dynamics, and then move to a local approach in order to target more precise questions regarding the system structure and dynamics.

Our brief introduction to control system dysregulation in Type 1/Type 2 diabetes was designed to illustrate how different types of dysregulation—of the same (in this case, metabolic) control circuit—might lead to markedly distinct clinical features. By analogy, we thus hypothesize that different psychiatric disorders might implicate the same prefrontal-limbic circuit, yet be dysregulated in different ways. Identifying those differences would be a first step towards a neurobiological model capable of explaining the clinical heterogeneity and dynamic trajectories of psychiatric signs and symptoms. In the next section, we extend this paradigm to the most basic evolutionary function of prefrontal-limbic regulation, *threat-detection*, and map its variation across a spectrum of human subjects across four studies (total *N* = 226), in order to identify a consistent picture of circuit regulation across acquisition modalities, tasks and analytic strategies.

## The Spectrum of Threat-Detection: From Hyper- to Hypo-Responsive

Historically, psychiatry disorders have been defined by statistical clustering of symptoms (DSM-5; American Psychiatric Association, 2013). More recent approaches, promoted by the United States National Institute of Mental Health (Research Domain Criteria, or RDoC), favor a dimensional approach across biologically defined criteria. Complicating matters is the fact that some psychiatric illnesses, such as schizophrenia, include significant clinical variation across multiple (emotional, cognitive and perceptual) domains. To avoid the need to consider interactions between domains, we therefore focus here upon a single prefrontal-limbic control circuit-based dimension, *threat-detection*, and assay the full range of threat-detection using control systems based approaches toward human neuroimaging. The spectrum ranged from most responsive to potential threat (which we hypothesize roughly aligns with psychiatry’s nomenclature of “clinical anxiety”) to least responsive to potential threat (which we hypothesize roughly aligns with psychiatry’s nomenclature of “sensation-seeking”). Because of the spectrum’s unidimensionality, it has the added advantage of providing more direct translation to genetic (rodent) studies (Stead et al., 2006; Davis et al., 2008; Simmons et al., 2012; Flagel et al., 2014) than do most psychiatric disorders, thereby contributing to our understanding of the underlying neurocircuitry.

### Trait Anxiety Study

In our first study, we tested *N* = 60 healthy individuals, and characterized their levels of trait anxiety using the *State-Trait Anxiety Inventory* (Spielberger, 2010). Subjects were presented with facial stimuli, both threat-related (angry and fearful-faces) and benign (neutral and happy faces), while being scanned with fMRI. Subjects also received ambulatory cardiac monitoring for 24 h. All 60 subjects’ fMRI scans were initially analyzed using activation maps (Mujica-Parodi et al., 2009a). We further analyzed a subset (*N* = 50) of artifact-free neuroimaging data using PSSI^4^, as well as obtaining autonomic regulation levels via principal dynamic modes (Tolkunov et al., 2010). Both low and high-trait anxious subjects showed strong amygdala activation to overly threatening stimuli; the differences occurred with respect to stimuli that were ostensibly benign. When presented with neutral faces, low-trait-anxious brains recognized the stimuli as “safe” and suppressed their amygdala responses accordingly. In contrast, high-trait anxious brains showed the same amygdala response whether faces were neutral or fearful/angry. This pattern suggests that anxiety might not be a disorder of *threat sensitivity*, but rather of *threat specificity*. Functional MRI time-series for subjects who were the least trait-anxious showed PSSI *β* ≈ 1 (pink-noise dynamics), which we described above as the signature of a negative feedback loop tuned optimally between excitatory and inhibitory components (Rădulescu and Mujica-Parodi, 2014). In contrast, fMRI time-series for subjects who were most trait-anxious scale showed PSSI *β* ≈ 0 (white-noise dynamics), the signature of a node unconstrained by other parts of the system (Rădulescu and Mujica-Parodi, 2014), Figure 3. These dynamics were distributed throughout the entire prefrontal-limbic circuit—as expected, since they are connected as part of a closed circuit—but with the trait anxious showing strongest dysregulation in the *pars triangularis*/Brodmann area 45, a subset of the IFG.

### Skydiver Study

Our first study showed that the brains of the “trait anxious” perceive non-threatening cues as threatening, and that dysregulation of prefrontal-limbic outputs has downstream autonomic consequences. For our second study (Mujica-Parodi et al., 2014), we probed the opposite end of the spectrum: those who perceived threatening cues as non-threatening. In order to characterize these subjects along the threat-response spectrum, we measured their subjective and physiological (cardiovascular, endocrine) responses to physical danger: first-time tandem skydives at 3.96 km with one full minute of free-fall. In many ways, skydiving provides an ideal experimental threat, as it is not only tests the body’s response to actual danger, but also has a highly standardized time-course that permits time-locked reproducibility across baseline/test sessions and subjects, and an ethical means of recruitment^5^. On the baseline day, hospitalized subjects received continuously cardiovascular monitoring using a holter ECG, were regularly assayed for cortisol, and at the end of the day received an MRI. On the test day, which occurred 1–2 weeks later, subjects repeated precisely the standardized protocol used during the baseline day, except that they jumped out of a plane and did not receive another MRI. Since our trait anxiety study suggested that the prefrontal-limbic system’s distinction between *threat* and *safety* was most clinically relevant, for the second study’s fMRI period we tasked subject’s brains with making the same distinction but changed the stimuli. This time, instead of affect-valent faces, subjects viewed a 16 s countdown that cued either an aversive (loud) or benign (soft) sound.

While the typical psychiatric construct of “sensation-seeking” (Zuckerman et al., 1972) distinguishes between those who do and do not seek out risky activities, our study was—by design—guided not by any diagnostic category but rather the spectrum of threat detection. All of our subjects independently chose to participate in a genuinely risky activity; what distinguished them, then, was the degree to which they *recognized* the risk, as measured by their subjective, endocrine and cardiovascular fear responses. As with the trait anxious, those who were more threat responsive showed greater amygdala activation. Importantly, relying solely upon amygdala activation, we might have erroneously concluded that individuals who showed less fear in response to the jump were more optimal prefrontal-limbic regulators than individuals who showed more fear. Yet the system-wide PSSI results told a fundamentally different story. This time, individuals who showed fear in response to the jump had *β* values closer to pink noise, the signature for a balanced—and therefore more efficient—control circuit. Instead, it was the individuals who remained impervious to the jump who showed *β* values closer to white noise—indicating weaker feedback throughout the circuit. PSSI identified here the same prefrontal-limbic regions that were previously implicated in the trait anxiety study; moreover, the area of most significantly disrupted dynamics was again localized specifically to the IFG, which correlated with cortical (structural) thinning of the same area. Behaviorally, more balanced IFG regulation was associated with greater accuracy in discerning ambiguous threat. In a behavioral task, skydivers who did not subjectively or physiologically recognize the skydive as threatening also showed higher thresholds in detecting morphed angry faces, consistent with results obtained using facial stimuli in our previous study. Together, our results made clear that the IFG must play a key role in processing ambiguous threat. However, what role that might be was unclear.

### Clinical Anxiety Study

Now having identified key nodes in the circuit via the global approach, we honed in on the circuit’s structure with the local approach, in order to understand the underlying basis for the PSSI dynamics. This time, we tested a population—Generalized Anxiety Disorder—for which responsiveness to potential threat we hypothesized to be a defining clinical feature. To this end, we used a fear generalization task (Greenberg et al., 2013a) in which subjects’ brains evaluated the salience of ambiguous stimuli, using stimuli perceptually similar to an abstract cue (rectangle) conditioned to indicate potential threat.

Healthy individuals showed activation of their vmPFC that were weakest in response to the most “threatening” cue (the conditioned stimulus), strongest in response to the most “safe” cue (the stimulus most perceptually dissimilar from the conditioned stimulus), with a linear gradient between the two poles (Greenberg et al., 2013b). The more anxious the subject, the more poorly the subject’s vmPFC discriminated between threat and safety cues, as shown by a decrease in the slope of the linear fit between the threat-to-safety poles. That the vmPFC activates in inverse proportion to perception of threat suggests that the vmPFC functions as the inhibitory component of the prefrontal-limbic circuit. Dynamic causal modeling of nodes (*vmPFC*, *IFG*, *amygdala*) identified via activation confirmed the connection from vmPFC to amygdala as inhibitory^6^.

Our measure of global circuit-wide feedback control, PSSI, once again identified the strongest difference between GAD and controls as dysregulation of the IFG. As with our study of trait anxiety, patients with Generalized Anxiety Disorder showed IFGs with PSSI *β*-values closer to white noise than healthy controls, with associated weaker autocorrelation within time-series of that area. These results, in response to the fear generalization task, were then independently confirmed in *N* = 65 subjects (43 subjects of whom participated in both studies) diagnosed with Generalized Anxiety Disorder (*N* = 45) as well as healthy controls (*N* = 20), who this time viewed naturalistic stimuli^7^. Remarkably, PSSI values yet again linked anxiety with dysregulation of the same IFG cluster (centered at MNI [−52, 20, 6] for Generalization task, MNI [−48, 22, 4] for naturalistic task), showing that our results were sufficiently robust to generalize across task design, and might apply in more “real-world” contexts.

A shift towards white noise suggests that the region, in patients, is less constrained by other parts of the meso-circuit; weaker autocorrelation indicates less “memory” within the time-series, a general feature of diminished feedback (Figure 3)^8^. We found that both functional (dynamic causal modeling) and structural (diffusion probabilistic tractography) connectivity support this hypothesis. Dynamic causal modeling demonstrated that subjects with more chaotic IFG dynamics showed weaker excitatory outputs from the IFG to the vmPFC. Likewise, brains whose IFG dynamics were most chaotic also had the weakest *uncinate fasciculus* (UF), a swath of long-range fiber bundles connecting the IFG and vmPFC with the amygdala. Given that the UF is the last white matter tract to develop (Lebel et al., 2008), we then investigated variation in network integration between the vmPFC and IFC in childhood temperament. Further neuroimaging of youngsters (*N* = 44, ages 3–5 years) using near-infrared spectroscopy revealed that children with less network integration within the prefrontal cortex had less emotional control, a risk factor for later psychopathology (Fekete et al., 2014).

## Making Sense of the Entire Spectrum: Lessons Learned and Future Directions

### Amending the Control Circuit to Include a Feedback Loop for Information Threshold

Taken together, our data converge to five preliminary conclusions, as summarized below and in Table 1.

**Table 1:** **Summary of experimental results for entire threat response spectrum**.

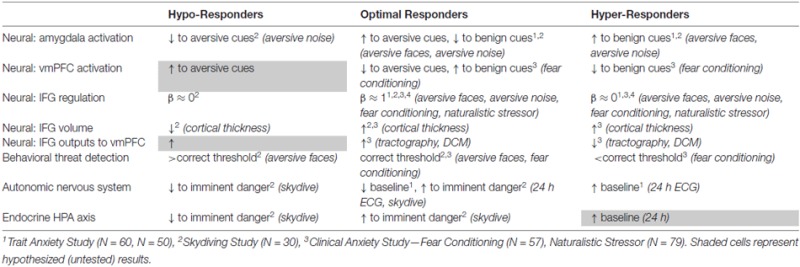

First, the amygdala and vmPFC provide dominant excitatory and inhibitory components, respectively, of the prefrontal-limbic circuit in assessing ambiguous threat, a relationship supported not only by reciprocal activation of the respective nodes across a gradient of threat detection but also by dynamic causal modeling.

Second, behavioral and neurobiological thresholds shift across the spectrum, with hyper-responders (e.g., trait and clinically anxious individuals) showing lower thresholds for detection of ambiguous threat and hypo-responders (e.g., “reckless” sensation-seekers) showing higher thresholds for detection of ambiguous threat.

Third, our computational modeling shows that optimally tuned negative feedback loops produce PSSI values in the pink noise range, and that shifts in PSSI values to white noise reflect dominant excitatory (chaotic) perturbations and/or diminished feedback within the system, with feedback affected by connection strength and lag.

Fourth, PSSI of fMRI time-series show that the IFG shows optimally tuned prefrontal-limbic “pink noise” regulation at the center of the threat detection spectrum, with both ends of the spectrum (hyper *and* hypo-responders) showing PSSI closer to white noise.

Fifth, our neuroimaging structural (volumetric, diffusion probabilistic tractography) and functional (dynamic causal modeling) connectivity data support *fundamentally different* sources of impaired circuit feedback at each end of the spectrum. Reckless individuals showed structural atrophy (cortical thinning) of the IFG, suggesting attenuation of the IFG’s function. Anxious individuals showed intact IFG volumes, but weakened output from this region to the vmPFC, suggesting that the IFG functions but fails to inform the rest of the circuit.

Confidence in the reliability of the five preliminary conclusions listed above is suggested by their robustness across multiple studies. Specifically, they remain consistent across clinical classifiers (measures designed to assess *trait anxiet*y across the healthy population, adult and developmental, as well as measures designed to assess *clinical anxiety*). They remain consistent across experimental designs, tasks and stimuli (*aversive faces*, *aversive noise*, *fear conditioning to electric shock*, and *naturalistic stimuli*). Finally, they integrate measured neural structural features (*volumetric analyses* and *tractography*), as well as downstream outputs to physiological (*autonomic*, *endocrine*) control circuits.

Since the only behavior that the two ends of the spectrum (hyper and hypo-responders) have in common is inaccurate threat assessment, we start from the working hypothesis that IFG regulation plays a key role in that inaccuracy, albeit in different ways that lead to opposite clinical features. In so doing, we adapt the canonical prefrontal-limbic circuit, in which the “low road” conveys lower-resolution information via a more direct route from the sensory organ to the thalamus, and then to the amygdala, while the “high road” conveys higher-resolution information via a more indirect route to the amygdala: first from the sensory organ to the thalamus, then to the sensory cortex, which provides additional sensory information, before looping back to modulate the amygdala as required. According to our revised model, the amygdala remains the primary excitatory component of the prefrontal-limbic control circuit, as our data confirm that the more a subject’s amygdala activates, the more responsive he is to ambiguous threat. Our fear-generalization data amend the LeDoux model to suggest that, of many candidate regions in the prefrontal cortex (*orbitofrontal cortex, medial prefrontal cortex, DLPFC, rostral anterior cingulate cortex, ventrolateral prefrontal cortex*) that have been implicated in inhibiting the amygdala, the only area that clearly tracked a safety (maximum activation) to threat (minimum activation) gradient was the vmPFC, whose connection to the amygdala was shown by dynamic causal modeling to be inhibitory.

Where our results most significantly diverge from the LeDoux model is in suggesting that the vmPFC’s inhibitory function receives crucial inputs from the IFG. The IFG’s role, both in our studies of threat detection (Mujica-Parodi et al., 2009a, 2014; Cha et al., 2016) as well as unrelated studies of meaning and perceptual ambiguity (Bozic et al., 2010; Rodd et al., 2012), imply that the IFG does not directly inhibit the amygdala, but rather may act as a set point for the amount of sensory information required to inform the vmPFC as to stimulus meaning (Roy et al., 2012). This function should be most evident when the potential for threat is ambiguous, an important feature of evolutionary environments in which true threats (e.g., predation) are almost always probabilistic and/or hidden. Neuroanatomically, the IFG is well positioned to mediate between the sensory cortex and the ventral prefrontal cortex for affective decision-making. It is connected with the sensory cortex, including the visual cortex, via an extensive associative white matter bundle (e.g., *inferior fronto-occipital fasciculus*)^9^. Implicated in affective cognition (Philippi et al., 2009), it is also connected to the primary emotion circuit (e.g., amygdala and ventral PFC) via the UF. Indeed, in our study, integrity of this white matter tract correlated with IFG time-series dynamics suggesting its participation in the larger control circuit (Cha et al., 2014, 2016). The IFG’s role as a convergence gate within the Information Loop is consistent with that of similar loops in the brain, such as the Hippocampal-VTA Loop (Lisman and Grace, 2005).

To determine if our revised circuit would result in key outcomes established by our four neuroimaging studies, we constructed a computational control systems model in MatLab Simulink v2016b (MathWorks, Natick, MA, USA), which interprets the structure shown in Figure 5 as a system of coupled differential equations. To modulate stimulus ambiguity, the signal (design matrix for our generalization task) was combined with different proportions of white noise. Raw sensory input to the thalamus contained all relevant frequencies, with sensory processing modeled as a band-pass filter, in which frequency cutoffs define the degree to which the complete signal is preserved. Therefore, we modeled the “low road” pathway to the amygdala, in which speed is optimized over accuracy, using a low-pass filter. The cortical “high road” starts with a wider range of frequencies, then further widens its filter to admit additional (higher) frequencies with every additional cycle through the visual processing stream. Both the “low road” and the “high road” include independent thresholds for threat amplitude, in the amygdala and vmPFC respectively, and converge on the comparator in the lateral amygdala, to either enhance or suppress central medial amygdala outputs. These outputs then feed forward to the reward circuit, hypothalamus, HPA-axis, and autonomic nervous system, and feed back to the lateral geniculate nucleus of the thalamus. Our circuit amends the canonical prefrontal-limbic system by adding an *informational threshold* in the IFG, which assesses its inputs in terms of whether required levels of sensory information are present in order to make a decision (operationally, this is done by using a Fast Fourier Transform (FFT) to quantify power amplitude for the signal). If the IFG identifies sufficient information, it outputs to the vmPFC, which in turn makes a threat determination of the data via its threat threshold. However, if the IFG informational threshold is not met, the data route back to the thalamus and sensory cortex for additional sensory processing, and continues to loop between the three areas (the Information Loop) until sufficient signal-to-noise ratios (SNR) are achieved. The model was constructed at the local field potential scale, in which dynamics represent populations of neurons firing. Synaptic transmission lag between brain regions was based upon a study of visual recognition in primates, suggesting signal delays of ~40 ms between regions (Tovee et al., 1994). To permit comparison of model outputs to fMRI data, neuronal signals were converted to the hemodynamic scale measured by fMRI via a balloon model for neurovascular coupling (Buxton et al., 2004).

**Figure 4:**
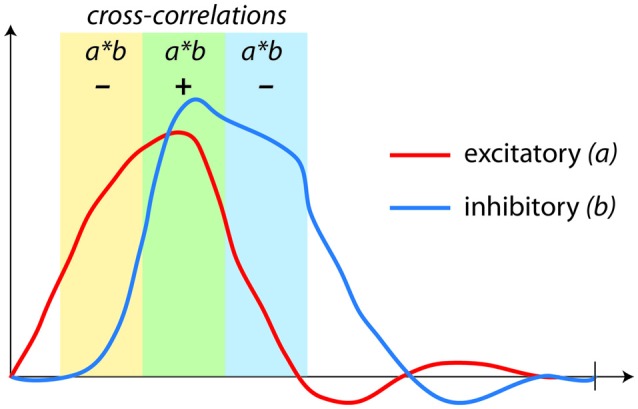
**Correlations (coupling) used in fMRI connectivity analyses are not capable of assessing regulation.** Here, a negative feedback loop with excitatory *(a)* and inhibitory *(b)* components produces time-series that appear to be either positively *or* negatively correlated, depending upon the stage of the dynamic process being assessed.

**Figure 5:**
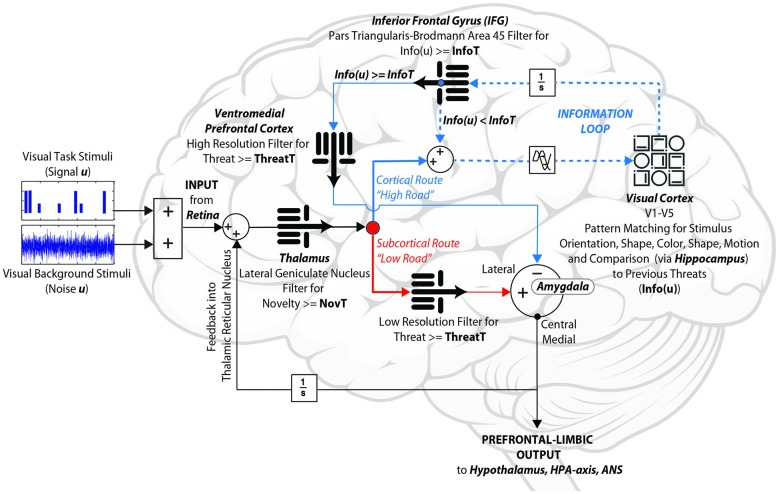
**Information-theoretic prefrontal-limbic control systems model.** Without relying upon *a priori* regions of interest, fMRI complexity analyses of four independent experiments converge to identify several key components implicated in the assessment of ambiguous (visual) threat: the *amygdala*, *visual cortex*, inferior frontal gyrus (IFG), and ventromedial prefrontal cortex (vmPFC). Our model integrates these within a control circuit constrained by known neuroanatomical connections. We hypothesize that the IFG gates the proposed *Information Loop* (*dotted line*), in order to determine whether there is sufficient signal-to-noise ratio (SNR) to make a provisional decision. Our data suggest that this loop appears to be disrupted for both hyper and hypo-responders. When the IFG’s *Information Threshold* (InfoT) is too high, the Information Loop cycles too many times, thereby failing to activate the vmPFC and thus allowing the subcortical route to dominate (*anxious*). On the other hand, when the IFG’s InfoT is too low, the Information Loop goes through too few cycles, thereby failing to process sensory data fully, and thus suppressing the amygdala outputs prematurely (*reckless*).

**Figure 6:**
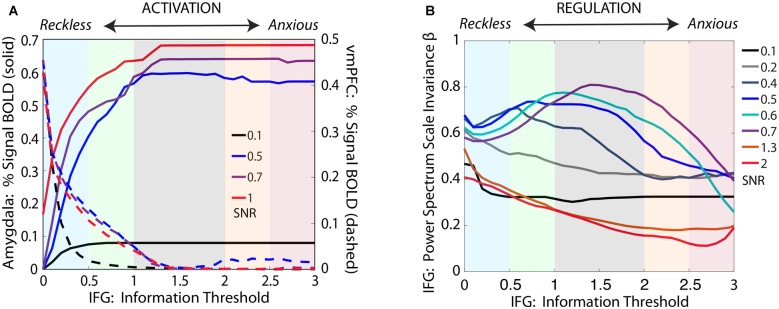
**Simulations for our computational model, shown in Figure 5, cohere with convergent experimental results across the threat-detection spectrum, for threat stimuli at varying levels of signal/noise (SNR). (A)** For ambiguous threat, activation for *amygdala* and vmPFC increase and decrease, respectively, across the reckless to anxious spectrum. **(B)** The IFG shows pink noise dynamics at the center of the spectrum, with shifts towards white noise dynamics at both ends of the spectrum. Importantly, our simulations demonstrate that the effect is not seen for either very low SNR (0.1–0.2) or very high SNR (1.3–2), but becomes evident only when the brain is challenged with ambiguous data (SNR = 0.05–0.07).

As shown in Figure 6, the model produces both activation (amygdala and vmPFC) and regulation (PSSI of IFG) results equivalent to those obtained across the spectrum of threat-detection, with effects most prominent when stimuli are maximally ambiguous between signal and noise. Of note, the Information Loop provides the brain with a mechanism by which it can process data in a Bayesian manner, by determining how much SNR is required in order to make provisional decisions in the face of incomplete information.

In starting with an analogy to Type 1/Type 2 diabetes, we made the point that the same control circuit can be dysregulated in more than one way, resulting in marked clinical differences. Based upon our neuroimaging results, our fifth preliminary conclusion suggested that in reckless individuals, the prefrontal-limbic circuit may be bypassing an impaired IFG, while in anxious individuals, the prefrontal-limbic circuit may be getting caught in the IFG loop and failing to exit. In fact, an important consequence of modulating the IFG set-point, is that setting it either too high or too low will result in the IFG providing less engagement with the rest of the circuit than is needed, but for different reasons. If the IFG threshold is too high, and requires so much sensory information that the IFG, thalamus, and sensory cortex exchange information between one another for too many cycles, in the meantime the coarse-grained excitatory pathway will dominate, with a greater likelihood for false positives in responding to ambiguous threat (as seen with anxiety). On the other hand, if the IFG threshold is too low, and requires too little information, the inhibitory pathway will suppress the amygdala without cycling back to the thalamus and sensory cortex for much-needed fine-grained sensory cortical data, with a greater likelihood for false negatives in responding to ambiguous threat (as seen with recklessness). Thus, the revised information-theoretic model can provide a data-driven description by which IFG regulation can produce the full spectrum of threat-detection: from anxious to optimal to reckless. As such, it provides a unifying neurobiological framework for understanding the relationship between populations, such as patients with anxiety disorders and “sensation-seekers” at risk for addiction, which are normally viewed as unrelated.

Traditional ways of thinking about anxiety and sensation seeking use “approach vs. avoidance” paradigms in response to novelty. In contrast, our focus is on how the brain interprets novelty and the inherent uncertainties that come with it, because we find that it better explains our data across the entire spectrum of hyper to hypo-responders. For example, the approach vs. avoidance paradigm cannot distinguish between the skydivers in our sample who jumped with recognition of the risks (optimal) vs. those who did not (reckless), in spite of the fact that these two cohorts deal with danger in ways that are likely to lead to very different outcomes. Moreover, while the standard assumption is that sensation-seekers seek out risks because they take exceptional pleasure in them, our sample of skydivers the individuals that self-identified as “sensation-seekers” (Zuckerman and Link, 1968) did not show stronger euphoric responses to the jump; either by subjective or endocrine (endorphin) criteria (Mujica-Parodi et al., 2014). As suggested by self-report, behavioral, neuroimaging and cortisol responses, they simply detected less risk and consequently experienced less fear. Finally, our construct is wholly neurobiological rather than questionnaire based. This has several advantages from the perspective of scientific rigor: it approaches mechanism rather than relying upon phenomenology, it permits more straightforward translational definition across species, and avoids many of the potential confounds inherent in self-assessment.

### Future Directions

Modeling and simulation provide evidence that a given paradigm is capable of producing observed data; however, to determine if the paradigm is the best solution as compared to other candidate solutions, it is necessary to conduct additional experimental research with cross-spectrum sampling. Our most significant limitation was that we developed our model as a process, as each new study forced us to refine and amend our hypotheses. Thus, while our studies are complementary, and therefore suggest a coherent story when combined as a whole, we lack a single study that used identical methods across the entire spectrum, thereby permitting direct comparison. Given lessons that we have learned, we can propose several candidate features and tools for such a comprehensive study.

Because we are interested in probing control circuit regulation, which governs both responsiveness to new stimuli as well as allostatic return to baseline, we start from the assumption that our neuroimaging will benefit from perturbation, and therefore some kind of stimuli. Our previous studies suggest that the circuit activates in response to ambiguous threat, and is sufficiently robust to be invariant to a specific set of stimuli or design. Our skydiving behavioral task (DeDora et al., 2011) had the advantage of dissociating response to *meaning* vs.* perceptual ambiguity*, while our generalization fMRI task had the advantage of using stimuli (geometric shapes) whose meaning (through conditioning) and perception (through percentage similarity) permit precise control in obtaining crisp gradients (amygdala and vmPFC in activation, IFG in regulation) in brain response. These might be fruitfully combined to provide one task capable of identifying behavioral responses to incomplete information, while permitting analysis the underlying neurobiology as the prefrontal-limbic circuit cycles during the information-acquisition phase. To assess global regulation of the circuit, using nonlinear complexity measures such as PSSI, one should aim for long (>10 min) tasks without preferred frequencies (Rubin et al., 2013). In addition, we have shown that fMRI acquisition parameters can be optimized specifically for dynamic fidelity (DeDora et al., 2016), thereby increasing the detection sensitivity and accuracy for both PSSI and control circuit analyses.

To avoid cognitive blocking, we might ambitiously consider even more targeted ways to probe the prefrontal-limbic circuit without conscious perception. We have previously shown that humans, like other animals, produce and detect alarm pheromones, defined as chemosensory cues that unconsciously signal potential threat to others of the same species. In an fMRI study and its replication (Mujica-Parodi et al., 2009b), we showed that sterile odorless sweat obtained from another subject while he/she underwent an emotional, but not physical, stressor—when inhaled reliably activates the amygdala. A follow-up behavioral study (Mujica-Parodi et al., 2009b), and its replication (Rubin et al., 2012), showed that when asked to distinguish between friend and foe for faces with ambiguous facial expressions, morphed between neutral and angry expressions, the alarm pheromone increased subjects’ threat-detection accuracy by 43%. Importantly, the pheromone reduced not only false negatives but also false positives. This behavioral effect was reflected by changes in subjects’ EEG event-related potentials: specifically, the *late positive potential* (LPP). The LPP measures neural response in the visual cortex (Hajcak et al., 2009). While the LPP is typically conceived of as related to “attention”, given the neurobiological and behavioral overlap between attention and emotional salience (Sander et al., 2003; John et al., 2016), as well as the LPP’s sluggish timing (160 ms post stimulus), it may be that the LPP is detecting the slower cortical route of the prefrontal-limbic control circuit, with recruitment of additional sensory (in this case, visual) information. While in response to exercise sweat (placebo), the LPP activated only in response to more threatening faces, fear sweat reliably triggered the LPP to all stimuli, which would thereby increase sensory processing for those that were more ambiguous (Rubin et al., 2012). As such, it may be the case that alarm pheromones improve cortical evaluation of potential danger by actively triggering the Information Loop between the IFG and sensory cortex. Moreover, if degree of cycling within the proposed Information Loop can be quantified by measuring LPP time-to-extinction following a stimulus event, it would open the door to more precise investigation of the timing of threat evaluation and its associated circuit dynamics. Further multi-modal neuroimaging along the threat-detection spectrum, in response to dynamic modulation of stimulus SNR, and following pharmacological manipulation of dopamine levels (potentially shifting IFG thresholds for SNR; Spitzer, 1997; Spitzer and Walter, 2003; Seamans and Yang, 2004; Krummenacher et al., 2010), would provide valuable directions for experimental testing of our model, and the dissociation of interactions between different loops (e.g., *IFG-thalamic reticular nucleus, IFG-vmPFC, amygdala-visual cortex, and amygdala-thalamus* (*mediodorsal, midline, pulvinar*)*-IFG*).

Our use of control systems to probe the threat-detection spectrum illustrates how clinical neuroscience may benefit from defining “dysregulation” within the context of closed-circuit feedback loops, which necessarily require characterization with nonlinear methods. As neuroimaging develops analytic approaches that move beyond the conceptual limits of activation or connectivity, clinical neuroscience will benefit by acquiring more powerful tools capable of probing how breakdown in homeostatic regulation may dissociate distinct mechanisms of disease.

## Author Contributions

LRM-P wrote the review article. LRM-P and JG conducted the computational modeling. LRM-P and JC reviewed the literature.

## Conflict of Interest Statement

The authors declare that the research was conducted in the absence of any commercial or financial relationships that could be construed as a potential conflict of interest.

## Footnotes

^1^Thus, diabetes is typically diagnosed using a glucose tolerance test, in which glucose values are measured 1–2 h following administration of a 75 g glucose bolus. In fact, the glucose tolerance test’s sensitivity and specificity as a diagnostic for metabolic dysregulation could be improved dramatically by measuring a continuous time-series of the system’s response and recovery from the bolus. For example, Graves disease produces dangerous levels of hyperglycemia, nearly double the amplitude of the glucose spike measured in diabetics. However, because Graves patients show full recovery by the 1–2 h mark employed by the glucose tolerance test, they thereby miss detection.

^2^For example, in the action potential the semi-permeable lipid bilayer membrane across an axon functions as the comparator. Inputs from each side of the membrane, manipulated by voltage-gated ion channels and/or diffusion, are compared across the membrane. The difference (the membrane “potential”) leads to a response output.

^3^These dynamics limit reliance upon not only amplitude-based (general linear model-type) analyses but also what is commonly known as “coupling” (cross-correlations) to identify regulation. This point can seem counter-intuitive because “regulation” and “dysregulation,” as referenced in the psychology and neuroimaging literature, have typically been understood as linear, in which two regions are involved in regulation (one excitatory and one inhibitory) are either directly or inversely coupled, in response to an experimental design that includes a task vs. control subtraction. However, if excitatory limbic and inhibitory prefrontal signals define a control circuit, then their activation should be strongly coupled with some small but finite lag. As shown by Figure 4, whether excitatory and inhibitory activation levels are directly or inversely correlated, depends upon whether one measures the beginning of the process (in which the excitatory component is elevated but the inhibitory level is not), the middle of the process (in which both excitatory and inhibitory components are elevated), or the end of the process (in which the excitatory component has been suppressed but the inhibitory component is still elevated). Since fMRI analyses are typically optimized for the activation levels rather than temporal features, one is likely to find both direct and inverse correlations between excitatory and inhibitory regions in the neuroimaging literature. These are not necessarily contradictory, but rather may be measuring the same circuit at different points of its regulation.

^4^The power spectrum scale invariance (PSSI) measure used in our earliest articles analyzed the first derivative of the time-series, which can be shown analytically to shift the slope of the raw time-series by a constant: (*β*_derivative_ = *β*_raw_ + 2. These early articles also used the equation *S(f) ∝ f^β^*, with the consequence that (*β*-values reported for human brain time-series were negative, unnecessarily complicating interpretation of their statistics. To permit straightforward comparison between all data sets, as well as to allow for interpretation of results using physical conventions for white/pink/brown noise, all results presented here are normalized to the raw (non-derivative) measure used in our later articles, and use the *S(f) ∝ f^−β^* equation so that reported *β* values are positive.

^5^For ethical reasons, we recruited subjects only from those who had independently contacted the skydiving school to schedule their first-time skydives. Therefore, we did not recruit subjects to skydive, but rather to permit us to collect measurements before, during, and after their skydives.

^6^What dynamic causal modeling (at the hemodynamic scale) interprets as grossly inhibitory, actually reflects excitatory projections to the amygdala enervating inhibitory neurons.

^7^Pilot episode of *Lost*, American Broadcasting Company television drama series.

^8^It is important to note that if optimal regulation can be characterized by pink-noise dynamics, dysregulation does not always mean that time-series are more chaotic, since de-optimization can occur in either direction (i.e., either too chaotic or too ordered). For example, in epilepsy, seizure foci are shifted towards *β* = 1, as dense arrays of short-range connections (which may reflect compensatory sprouting in response to neuronal damage) locally constrain dynamics (Nedic et al., 2015). Figure 1 provides another example in which the particular type of dysregulation (Type 1 diabetes), in this case caused by weak inhibition, produces time-courses that are too stiff rather than too chaotic.

^9^Human data informatics from the MGH-USC Human Connectome Project show that the strongest connection from the *left BA45/pars triangularis* is to the *right BA45/pars triangularis*, which in turn connects to occipital regions (http://www.humanconnectomeproject.org/informatics/relationship-viewer/).
